# Inaccuracy in the Article “Scrambler Therapy Treatment: The Importance of Examining Clinically Meaningful Improvements in Chronic Pain and Quality of Life”

**DOI:** 10.1093/milmed/usaa089

**Published:** 2020-05-22

**Authors:** Giuseppe Marineo, Giuseppe Marineo

**Affiliations:** Delta Research and Development, University of Rome Tor Vergata, Via di Mezzocammino 85, Rome 00127, Italy

*Dear Editor*:

**Inaccuracy in the Article “Scrambler Therapy Treatment: The Importance of Examining Clinically Meaningful Improvements in Chronic Pain and Quality of Life”**

I read with great interest of the article “Scrambler Therapy Treatment: The Importance of Examining Clinically Meaningful Improvements in Chronic Pain and Quality of Life.” I share the intent of the article, and thank the authors for their work.

Although the study is well conceived, and undoubtedly useful, it unfortunately does not appear to comply with the standard methods of use that registered in the procedure FDA 510(k) # K142666. These standards have also been the object of a recent scientific publication that provides the scientific and medical community with further clarification and documentation on how to avoid or reduce bias in clinical studies on Scrambler Therapy.

I must also clarify that only the first generation of Scrambler Therapy devices marketed in the USA uses the commercial brand name “Calmare” (510 (k) # K081255).

It was during this initial phase of distribution in the USA that information on the correct use of the device were not fully aligned with the original specifications. This depended on the fact that there were many independent attempts to deviate from the official standards (the original standards provided with the license and registered with the health authorities), aimed at simplifying the placement and regulating procedures, the duration of the treatment period, and consequently, the cost and the usage difficulties. However, although these attempts were justified from a commercial point of view, the results produced have always been extremely disappointing.

Objectively, these elements of deviance created by the spread of official and nonofficial information and methods, also present in many independent publications, have undoubtedly created a great deal of uncertainty and confusion in the medical and scientific community. I presume this is one of the reasons why the authors were unable to use adequate information to conduct the study correctly.

More specifically, the proper use of Scrambler Therapy, being operator-dependent, allows only for a partial double-blind or single-blind trial design. Attempts to do a complete double-blind clinical trial automatically cause substantial changes in the standard treatment protocol, which requires substantial patient interaction to determine proper placements of electrodes and intensity of treatment. These changes prevent the operator to follow the normal procedures registered in the health care authorizations and can erase or significantly reduce the efficacy of the treatment, consequently invalidating the scientific data. In addition, the nonconformity of the placement and interactive regulation procedures, in some conditions makes an increase in pain highly likely, instead of completely suppressing it in each individual treatment.

As regards the device used as sham, it should be noted that for correct regulation, the stimulation must be perceived by the patient under the electrodes only. Consequently, an “ineffective” perception threshold does not exist. For the same reason, the electrodes should be positioned well away from the pain area to avoid spurious interference.

I would once again like to thank the authors for their important contribution, and I hope the clarification that I have provided is useful for future clinical studies.

## DECLARATION OF CONFLICTS OF INTEREST

The author developed the Scrambler Therapy basic and applied research and is the patent owner of the ST technology.

Giuseppe Marineo, researcher, ScD

Delta Research and Development, University of Rome Tor Vergata, Via di Mezzocammino 85, Rome 00127, Italy

